# Water-Soluble Lignins from Different Bioenergy Crops Stimulate the Early Development of Maize (*Zea mays*, L.)

**DOI:** 10.3390/molecules201119671

**Published:** 2015-11-05

**Authors:** Davide Savy, Vincenza Cozzolino, Giovanni Vinci, Antonio Nebbioso, Alessandro Piccolo

**Affiliations:** Interdepartmental Research Center of Nuclear Magnetic Resonance for the Environment, Food Processing and New Materials (CERMANU), Via Università 100, Portici 80055, Italy; cozzolino.vincenza@libero.it (V.C.); giovannivinci83@libero.it (G.V.); antonio.nebbioso@unina.it (A.N.)

**Keywords:** water-soluble lignins, eucalyptus, cardoon, black poplar, ^31^P-NMR, ^13^C-CPMAS-NMR, infrared spectrometry, maize seed germination, maize growth, plant biostimulants

## Abstract

The molecular composition of water-soluble lignins isolated from four non-food bioenergy crops (cardoon CAR, eucalyptus EUC, and two black poplars RIP and LIM) was characterized in detail, and their potential bioactivity towards maize germination and early growth evaluated. Lignins were found to not affect seed germination rates, but stimulated the maize seedling development, though to a different extent. RIP promoted root elongation, while CAR only stimulated the length of lateral seminal roots and coleoptile, and LIM improved only the coleoptile development. The most significant bioactivity of CAR was related to its large content of aliphatic OH groups, C-O carbons and lowest hydrophobicity, as assessed by ^31^P-NMR and ^13^C-CPMAS-NMR spectroscopies. Less bioactive RIP and LIM lignins were similar in composition, but their stimulation of maize seedling was different. This was accounted to their diverse content of aliphatic OH groups and S- and G-type molecules. The poorest bioactivity of the EUC lignin was attributed to its smallest content of aliphatic OH groups and largest hydrophobicity. Both these features may be conducive of a EUC conformational structure tight enough to prevent its alteration by organic acids exuded from vegetal tissues. Conversely the more labile conformational arrangements of the other more hydrophilic lignin extracts promoted their bioactivity by releasing biologically active molecules upon the action of exuded organic acids. Our findings indicate that water-soluble lignins from non-food crops may be effectively used as plant biostimulants, thus contributing to increase the economic and ecological liability of bio-based industries.

## 1. Introduction

Waste-derived materials have nowadays assumed great importance as valuable source of marketable chemicals and goods. Used cooking oil, for example, may be employed as starter for the production of bio-diesel [1] or lubricants [2], while citrus peels may be processed to obtain pectin [3], succinic acid [4] or D-limonene [5]. For a long time lignin residues from biorefineries and paper mills has been considered a waste, and consequently burnt or otherwise disposed of [6]. Conversely, researchers have recently devoting been their efforts to optimizing lignin extraction, in order to obtain different kind of chemicals [7].

Among the various methods devised for lignin isolation, that employing alkaline solutions allows one to extract water soluble lignins containing well fragmented and oxidized phenols and a large content of carboxyl and hydroxyl functional groups [8]. Lignin hydrolysis and water solubility is further improved by adding hydrogen peroxide (H_2_O_2_) to the reaction mixture [9], thus facilitating the disruption of plant cell walls, and enhancing the separation from cellulose. Water-soluble lignins are reported to act as plant biostimulants, thus concomitantly improving plant growth and stress resistance with a bioactivity similar to that of HUmic-Like Substances (HULIS) [10,11,12]. The heterogeneous humic substances (HS) and HULIS originate from the microbial decay of plant and animal biomass and are ubiquitous in the environment [13]. They may positively affect plant physiology and growth by modifying the activity of plasmalemma H^+^-ATPase, and, thus, enhancing the elongation and proliferation of secondary roots in maize (*Zea mays*, L.) [14]. The addition of HS or HULIS, may also induce a re-distribution of bioactive molecules, such as cytokinins and polyamines, in shoots and roots, with a consequent increase of shoot development in watermelon (*Cucumis sativus*, L.) [15]. Moreover, maize plants added with lignosulphonates-humates were shown to contain more glucose and fructose in the whole plant and consequently to increase both root and leaf growth [16], while tomato (*Lycopersicon esculentum*, L.) growth was significantly improved by treating seedlings with phenolic mixtures from spruce (*Picea abies*, L.) bark [17]. Furthermore, HS and HULIS are recognized to protect plants from either biotic or abiotic stresses [18,19].

Despite the large amount of lignin-rich waste materials, this precious photosynthetate is not yet sufficiently proved to act as a sustainable biostimulant for an intensified agriculture. The aim of this work was to verify the bioactivity towards maize (*Zea mays*, L.) germination and early growth of fully characterized water-soluble lignins from four different biomasses (cardoon, eucalyptus and two black poplars) and to attempt a structure-activity relationship.

## 2. Results and Discussion

### 2.1. Extraction Yields

The yield of lignin recovered from CAR and RIP was 3.6%, whereas it was 13.6% and 1.6% from LIM and EUC, respectively. The standard deviation was lower than 0.2 for all the biomasses. The low extraction yield was attributed to either a specific lignin content or an advanced degradation under the alkaline H_2_O_2_ treatment [20]. However, since these materials are recovered in large amounts from wastes of biorefineries or paper mills, low yields may have a negligible importance as compared to the productive potential of the bio-based industries [7,21].

### 2.2. DRIFT-IR Spectra

**Figure 1:**
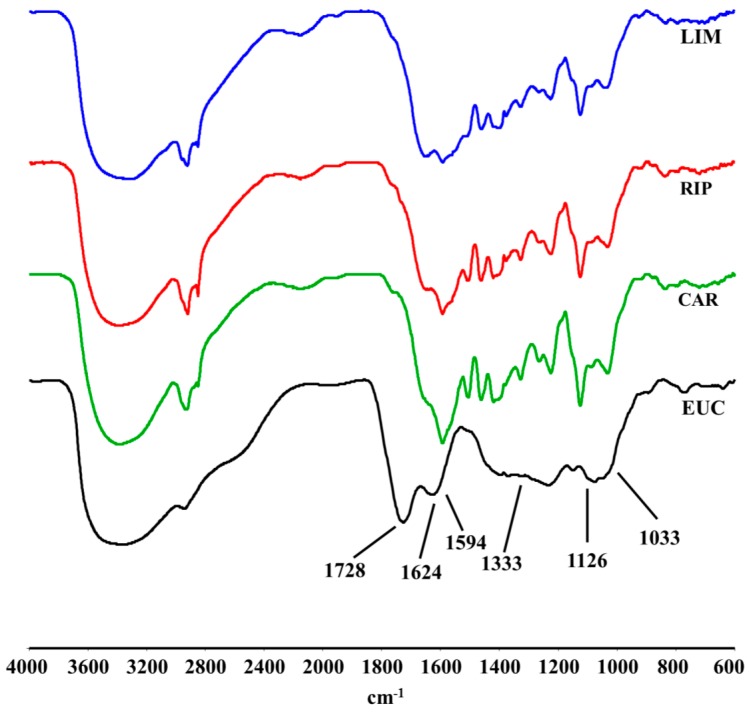
DRIFT-IR spectra of water-soluble lignins isolated from cardoon (CAR), eucalyptus (EUC), black poplar grown in Ripiti (RIP) and black poplar grown in Limatola (LIM) by an alkaline oxidative extractant.

The DRIFT-IR spectra of the extracted lignins are shown in Figure 1. Signals around 3392–3360 cm^−1^ were attributed to the OH vibrations, while those around 2930 and 2850 cm^−1^ were due to C-H stretching in alkyl chains [22]. The signals, around 1590, 1500 and 1420 cm^−1^ were assigned to the vibrations of aromatic rings [23], while those at about 1330, 1265, 1125 and 1030 cm^−1^ is associated to specific vibrations in lignin molecules. The absorption around 1330 cm^−1^, in fact, is due to vibrations of condensed syringyl and guaiacyl rings bound through the 5-position [24], while the ring breathings for both these monolignols are located around 1265 and 1125 cm^−1^, respectively. Additional signals related to the vibrations of lignin molecules were recognized around 1030 cm^−1^, and attributed to aromatic C-H in-plane deformation (G > S) [20]. Finally, the band around 1730 cm^−1^ is assigned to C=O stretching in carbonyl and esters function, as in conjugated aromatic units or residual carbohydrate structures. The latter hypothesis is also supported by the presence of the band around 1080 cm^−1^, due to C-C-O stretching in secondary alcohols [25], and by NMR results.

### 2.3. ^13^C-CPMAS-NMR Spectroscopy

**Figure 2:**
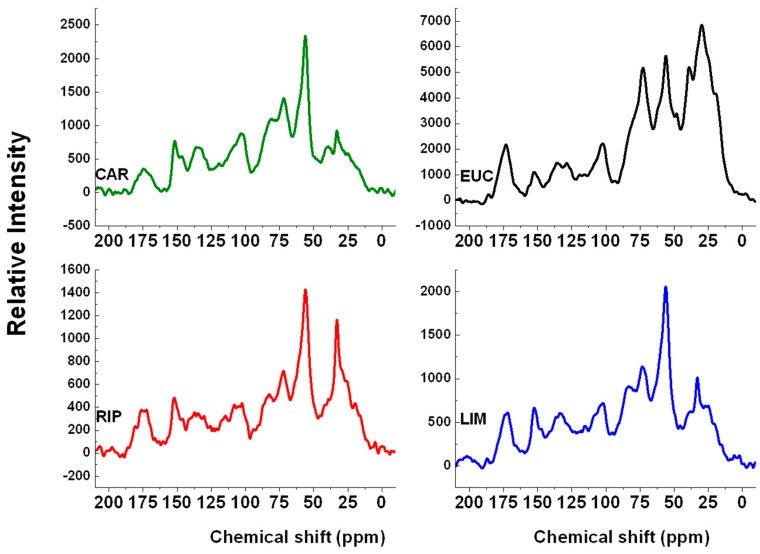
^13^C-CPMAS-NMR spectra of water-soluble lignins isolated from cardoon (CAR), eucalyptus (EUC), black poplar grown in Ripiti (RIP) and black poplar grown in Limatola (LIM) by an alkaline oxidative extractant.

The solid-state NMR spectra revealed signals typical of lignin molecules, such those at 152 and 135 ppm in the aromatic range (Figure 2). In particular, the former signal was assigned to the resonance of C_3_ and C_5_ in ethers of syringyl units, whereas the latter one was typical of C_1_ in both syringyl and guaiacyl monomers [26]. Moreover, the signal at 56 ppm is attributed to methoxy carbons, while those around 39, 32 and 29 ppm are due to alkyl carbons [20]. The occurrence of residual carbohydrates in all lignin extracts from biomass was revealed by the signals at 172, 103, 81 and 72 ppm, which accounted for acetyl groups, anomeric carbons and hemicellulose oligomers [27]. The carbon distribution over the spectral chemical shift ranges indicated that the carboxyl carbon was comparable in all biomass extracts, being 7% in both RIP and LIM, 6% in EUC and 4% in CAR (Table 1). Conversely, the aromatic carbon was significantly lower in EUC than in the rest of lignins, while carbohydrate and methoxy carbons, in the 112–46 ppm range, were similar in all extracts (Table 1). In the case of EUC, the aliphatic carbon was much larger (40%) that for the other lignins, which accounted to 19%, 25% and 19% for CAR, RIP and LIM, respectively. The spectral carbon distribution allows the calculation of hydrophobicity (HB), hydrophilicity (HI), and the HB/HI index. The greater the hydrophobicity of a complex organic material, the larger is its potential interaction with low polarity compounds and the smaller is with those of higher polarity [28]. The HB/HI index indicates that the extent of oxidation in lignin extracts is different according to the biomass of origin. The most hydrophobic character was shown by EUC and RIP extracts, in the order, whereas both LIM and CAR lignins showed progressively more hydrophilic properties and, thus, lower HB/HI values (Table 1).

**Table 1:** Carbon distribution (%) in different chemical shift regions (ppm) in ^13^C-CPMAS-NMR spectra of lignins from cardoon (CAR), eucalyptus (EUC), black poplar grown in Ripiti (RIP) and black poplar grown in Limatola (LIM).

Lignin Extracts	Chemical Shift Regions					Indexes		
	187–162 (COOH)	162–112 (aromatic-C)	112–93 (anomeric-C)	93–46 (C-O, C-N)	46–0 (Alkyl-C)	HB	HI	HB/HI
CAR	4	20	11	48	17	37	63	0.59
EUC	6	11	7	38	38	49	51	0.96
RIP	7	19	9	40	25	44	56	0.79
LIM	7	20	10	44	19	39	61	0.64

HB = (162–112) + (46–0); HI = (187–162) + (112–46); HB/HI = (162–112) + (46–0)/(187–162) + (112–46).

### 2.4. ^31^P-NMR Spectroscopy

Derivatization of lignin extracts with a ^31^P marker, such as 2-chloro-4,4,5,5-tetramethyldioxaphospholane (CTMP) allowed to study the quality and quantity of OH functions in lignins by ^31^P liquid-state NMR spectroscopy. Due to the broad spectral range of ^31^P spectra and inherent high spectral resolution, it is possible to accurately identify the various types of OH-carrying molecules in ^31^P-labelled in natural samples [29,30,31,32].

**Figure 3:**
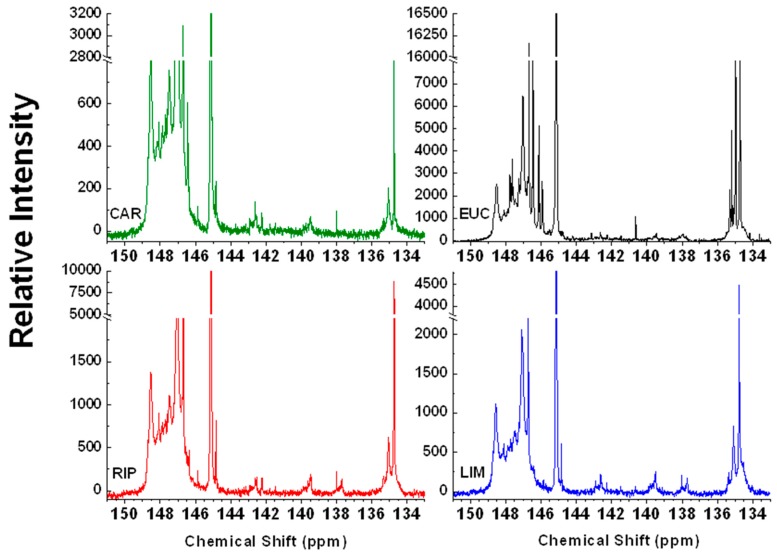
^31^P-NMR spectra of water-soluble lignins isolated from cardoon (CAR), eucalyptus (EUC), black poplar grown in Ripiti (RIP) and black poplar grown in Limatola (LIM) by an alkaline oxidative extractant and derivatized with 2-chloro-4,4,5,5-tetramethyldioxaphospholane.

The spectra of phosphytilated lignins are shown in Figure 3, while the quantitative content of different groups identified in ^31^P-spectra are reported in Table 2. The content of carboxyl OHs (135.3–133.7 ppm) [30] was variable among samples, with EUC and CAR containing the highest and lowest amount of COOH (1.41 and 0.42 mmol·g^−1^ of lignin), respectively, and with LIM and RIP showing similar content of acidic functionalities (Table 2). The guaiacyl (G) and syringyl (S) OH groups [31] were greater in CAR and LIM than in EUC and RIP, while the OHs in *p*-hydroxyphenyl (H) moieties were generally small and even undetected in CAR. This absence in CAR was expected, since lignin of cardoon is essentially composed by S- and G-type molecules, as reported earlier [33]. Furthermore, the OHs in condensed lignin dimeric units (142.8–141.7 ppm) were comparable in EUC and CAR, while they were much lower in the two poplar-derived lignins (Table 2). The amount of total phenolic units in ^31^P-spectra of lignin extracts were relatively small and lower than the semi-quantitative 20% of, aromatic carbon found by ^13^C-CPMAS-NMR spectroscopy (Table 1). This discrepancy may account for a large presence of S- and G-compounds as ethers (mainly β-O-4′ dimers), rather than free phenols, which cannot react with the CTMP derivatizing agent and detected by ^31^P-NMR. In fact, the β-O-4′ linkages are degraded under alkaline oxidative conditions only to a certain extent, and persist as ethers in lignin, as shown earlier by 2D-NMR studies [34,35]. Conversely, the small amount of phenols in EUC agrees with results by ^13^C-CPMAS (Table 1), since the large reactivity of S-molecules towards alkaline H_2_O_2_ may have decreased significantly the content of aromatic moieties [36,37], thus suggesting a larger EUC fragility to oxidation than for cardoon and black poplar biomasses. Finally, all the biomasses showed a large content of free hydroxyalkyl functionalities (150.8–146.3 ppm) [30], with a decreasing amount in going from CAR to RIP, LIM and EUC, in that order.

**Table 2:** Structural assignment, chemical shift ranges, and integrated amount (mmol·g^−1^ of lignin) of main signals in ^31^P-NMR spectra of lignins isolated from cardoon (CAR), eucalyptus (EUC), black poplar grown in Ripiti (RIP) and black poplar grown in Limatola (LIM) by alkaline oxidative extraction.

Chemical Shift (ppm)	Assignment	CAR	EUC	RIP	LIM
135.6–133.7	Carboxyl groups	0.42	1.41	1.02	1.23
138.6–136.9	H-type molecules	ND	0.07	0.04	0.07
140.2–138.4	G-type molecules	0.11	0.04	0.07	0.14
142.8–141.7	Condensed (4-O-5′,5-5′) lignin dimers	0.14	0.11	0.04	0.04
143.7–142.2	S-type molecules	0.14	0.04	0.07	0.11
143.7–136.9	Total Phenolic Content	0.39	0.25	0.21	0.35
150.8–146.3	Aliphatic OH	6.76	3.94	5.42	4.68

ND: Not Detectable.

### 2.5. Thermal Analyses

**Figure 4:**
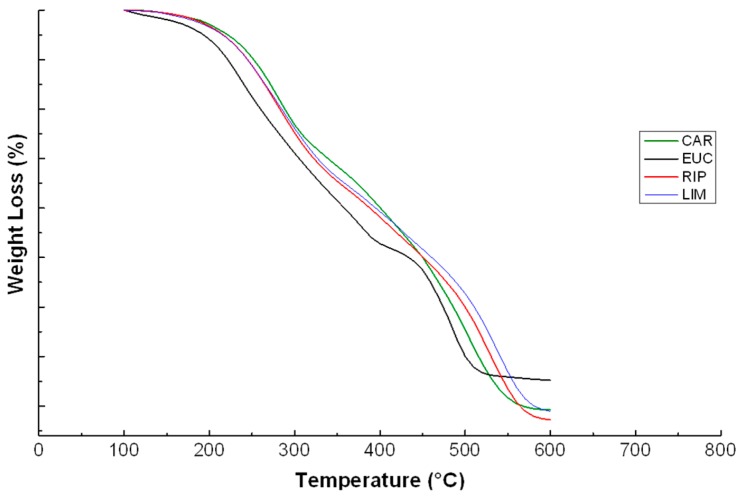
Thermograms of water-soluble lignins isolated from cardoon (CAR), eucalyptus (EUC), black poplar grown in Ripiti (RIP) and black poplar grown in Limatola (LIM) by an alkaline oxidative extractant.

**Figure 5:**
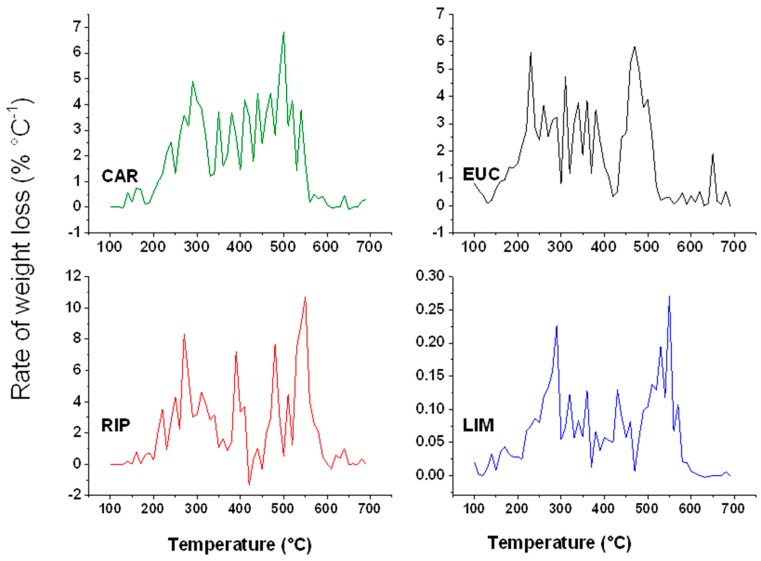
DTG curves of water-soluble lignins isolated from cardoon (CAR), eucalyptus (EUC), black poplar grown in Ripiti (RIP) and black poplar grown in Limatola (LIM) by an alkaline oxidative extractant.

The TGA curves for lignins are shown in Figure 4. The extracts from cardoon and the two black poplars displayed very similar thermal behaviour. In contrast, EUC lignin showed a more rapid weight loss, thus indicating that such a thermal weakness may be related to an advanced depolymerization caused by the isolation procedure [20]. This finding is in line with NMR results (Table 1 and Table 2), which suggested that EUC lignin had reacted with the extractant more than CAR, RIP and LIM. More information on the type of molecules progressively degraded during TGA are obtained by its first derivative, and as shown by the relative DTG curves (Figure 5). Peaks within 220–300 °C range are commonly attributed to decomposition of lignin side-chain and hemicellulose residues [38,39], while those in the 320–350 °C have been interval are ascribed to degradation of phenols and C-C bonds between monolignols [20,40]. Finally, aromatic ring oxidation occurs at about 400–500 °C [41].

### 2.6. Germination of Maize Seeds and Early Growth

The response of the maize root apparatus upon treatments with increasing lignin concentrations was studied by measuring the length of both radicle and lateral seminal roots (LSR), while the elongation of coleoptile provided information about the effect of lignin application on the plant epigean part (Table 3, Table 4 and Table 5). Raw data for such experiments are reported in the Tables S1–S4 for lignin isolated from CAR, EUC, RIP and LIM, respectively. While lignin additions did not affect the rate of maize seeds germination (data not shown), treatments with lignins at different concentrations influenced the growth of maize plantlet. In order to compare results among lignins from different biomasses, lignin treatments were normalized in respect to control, whose value was set to 100 (Table 3, Table 4 and Table 5).

**Table 3:** Length (cm) of radicle roots for maize seedlings treated with different concentrations (ppm) of CAR, EUC, RIP and LIM water-soluble lignins.

Lignin Concentration (ppm)	CAR	EUC	RIP	LIM
0—Control	6.5 (100) ^a^	10.8 (100) ^a^	5.5 (100) ^a^	6.4 (100) ^a^
0.1	5.3 (82) ^a^	10.8 (100) ^a^	6.7 (122) ^a,b^	6.4 (101) ^a^
1	6.3 (98) ^a^	10.6 (99) ^a^	5.9 (107) ^a,b^	7.4 (116) ^a^
10	7.0 (108) ^a^	9.5 (88) ^a^	6.4 (117) ^a,b^	6.3 (99) ^a^
100	4.9 (76) ^a^	10.5 (96) ^a^	6.8 (124) ^b^	7.5 (118) ^a^

Values in parenthesis are normalized to control values set at 100. Different letters indicate significant differences in column at 0.05 probability level, as revealed by the Tukey’s test.

**Table 4:** Length (cm) of lateral seminal roots for maize seedlings treated with different concentrations (ppm) of CAR, EUC, RIP and LIM water-soluble lignins.

Lignin Concentration (ppm)	CAR	EUC	RIP	LIM
0—Control	5.9 (100) ^a^	19.8 (100) ^a^	8.2 (100) ^a^	8.7 (100) ^a^
0.1	6.5 (111) ^a,b^	19.3 (98) ^a^	11.2 (135) ^b^	8.9 (102) ^a^
1	7.0 (118) ^a,b^	18.6 (94) ^a^	7.9 (96) ^a^	10.2 (118) ^a^
10	8.3 (140) ^b^	16.0 (81) ^a^	9.9 (120) ^a,b^	9.3 (107) ^a^
100	5.7 (97) ^a^	20.8 (105) ^a^	10.2 (123) ^a,b^	8.9 (102) ^a^

Values in parenthesis are normalized to control values set at 100. Different letters indicate significant differences in column at 0.05 probability level, as revealed by the Tukey’s test.

**Table 5:** Length (cm) of coleoptiles for maize seedlings treated with different concentrations (ppm) of CAR, EUC, RIP and LIM water-soluble lignins.

Lignin Concentration (ppm)	CAR	EUC	RIP	LIM
0—Control	3.6 (100) ^a^	8.5 (100) ^a^	5.1 (100) ^a^	5.2 (100) ^a^
0.1	5.5 (150) ^b,c^	8.9 (104) ^a^	5.4 (102) ^a,b^	5.7 (110) ^a,b^
1	5.3 (146) ^b,c^	8.8 (104) ^a^	5.5 (104) ^a,b^	5.5 (106) ^a,b^
10	6.3 (172) ^c^	8.1 (95) ^a^	5.6 (107) ^a,b^	5.4 (104) ^a,b^
100	4.6 (127) ^a,b^	8.4 (98) ^a^	5.9 (113) ^b^	6.0 (117) ^b^

Values in parenthesis are normalized to control values set at 100. Different letters indicate significant differences in column at 0.05 probability level, as revealed by the Tukey’s test.

Among lignin extracts, only RIP showed an enhancement of radicle elongation at all concentrations studied (Table 3), with 100 ppm as the most stimulating concentration (24% more than the control). The length of lateral seminal root (LSR) was increased only by the CAR and RIP lignin extracts (Table 4). However, a significant LSR improvement in maize seedlings (35% more than control) was obtained already at 0.1 ppm of RIP, whereas 10 ppm were required for a significant effect (40% more than the control) of the CAR extract (Table 4).

The effect of water-soluble lignins on coleoptile length of maize plantlets was evident for all extracts except for the EUC lignin (Table 5). Lignins extracted from cardoon (CAR) and the two black poplars (RIP and LIM) significantly increased maize coleoptile growth over the control at all concentrations. In particular, CAR was already effective (50% more than control) at the lowest concentration (0.1 ppm), while seedling growth reached 72% more than control with the 10 ppm treatment. A somewhat smaller stimulation of coleoptile growth (27% more than control) was shown by the addition of the 100 ppm solution (Table 5). Both RIP and LIM lignin extract were similar in significantly increasing the coleoptile elongation of about 15% more than control only at a concentration of 100 ppm (Table 5).

The largest elongation provided by the CAR lignin on both LSR and coleoptile can be related to its largest content of aliphatic hydroxyl functions (Table 2), and its smallest hydrophobicity, HB/HI index, and carboxyl carbons (Table 1). The lignins from RIP and LIM, which behaved similarly in providing a smaller bioactivity than CAR towards coleoptile elongation, showed also comparable chemical features. In particular, carboxyl groups, total phenolic and aliphatic OH functions were similarly more abundant in RIP and LIM than in CAR (Table 2), while hydrophobicity and HB/HI index were larger (Table 1). However, lignin from RIP greatly promoted root growth, while LIM did not (Table 4). This may be related to a different conformational stability conferred by a concomitant larger content of aliphatic OH groups and smaller content of S- and G-type molecules in the former than in the latter (Table 2).

The difference in bioactivity of the different lignin extracts should be accounted to their molecular composition and the consequent stability of their conformational structures in water. Water-soluble lignins isolated by alkaline peroxidation from non-food biomasses were shown to behave as supramolecular associations inasmuch as humic natural organic matter [10,13]. The lignin molecules are held in metastable conformational structures by hydrophobic and hydrogen bonds which may be easily disrupted by action of organic acids [28,42]. Thus, when lignin extracts interact with the organic acids exuded by vegetal tissues, their original supramolecular conformation may be altered as a function of their molecular composition, thereby releasing bioactive molecules [10,14,43].

This humic-like structural behaviour of water-soluble lignins may be then related to the observed differences in bioactivity on maize seedlings. It is plausible to attribute the lack of bioactivity of the EUC extract at any concentration to its largest hydrophobicity provided by the great number of poorly hydroxylated long-chain alkyl molecules, which make the EUC conformational structure too much rigid to be altered and become a source of hydrophilic bioactive molecules. Conversely, the CAR lignin, and by a lower extent the RIP and LIM extracts, showed a more hydrophilic character, and therefore, they were more prone to release bioactive molecules from an easily disrupted conformational structure in water. Savy *et al.* [10] have earlier related the bioactivity towards maize development and growth of well-fragmented water-soluble lignins from two different energy crops (giant reed and miscanthus) to both the physical-chemical and molecular characteristics of lignin extracts. Moreover, the different bioactivities of the two lignins were accounted to both their apparent molecular size, as assessed by both HPSEC and DOSY-NMR spectra, and content of free phenolic units in the two extracts.

The different lignin-derived free phenols in the biomass extracts may well be the bioactive molecules, whose release from the extract supramolecular assembly may be promoted by the organic acids. In fact, water-soluble phenolic mixtures may interact with the plant cell walls and affect plant physiology, thus inducing morphological changes in roots and shoots. Lignosulfonates-humates were shown to boost the activity of enzymes involved in nitrogen organication and photosynthesis (namely, GS, GOGAT and rubisco) in maize plantlets and increase concentration of proteins, glucose and fructose in both roots and leaves [16]. Furthermore, addition of lignosulfonates to bean (*Phaseolus vulgaris*, L.) determined an increase in the mitotic index of treated plants and a consequent larger total dry weight and germination capacity [44]. Indeed, higher transpiration rates and CO_2_ assimilations, as well as larger amount of photosynthetic pigments were noted after soil and foliar application of spruce-derived polyphenols on sunflower (*Helianthus annuus*, L.) plants, thus leading to an increased dry weight of roots, stems, leaves, and seeds [45]. Phenolic mixtures are also known to possess hormone-like activity. In fact, phenol-based biostimulants display both auxin (IAA)- and gibberellin (GA)-like activity [10], and also solutions of pure phenolic acids may exert similar effects [46,47]. In fact, while *p*-hydroxybenzoic, vanillic and syringic acids acted as GA-like molecules [46], gallic, protocatechuic and phenylacetic acids revealed an IAA-like effect [47] in specific bioassays on watercress (*Lepidium sativum*, L.) and lettuce (*Lactuca sativa*, L.), respectively.

## 3. Experimental

### 3.1. Biomasses

Cardoon (*Cynara cardunculus*, L.—CAR) was cropped on September 2012 at the University of Naples experimental farm in Bellizzi (Salerno, Italy); eucalyptus (*Eucalyptus camaldulensis*, Dehnh.—EUC) was harvested on March 2012 from the nearby experimental farm of Eboli (Italy); the two black poplars (*Populus nigra*, L.) were cropped on March 2012 along the Ripiti (Salerno, Italy) (RIP) and the Limatola (Benevento, Italy) (LIM) creeks.

### 3.2. Alkaline Oxidative Hydrolysis

Lignin isolation was carried out as earlier described by Sun *et al.* [48]. Briefly, each biomass (10.0 g) was added with a 2% H_2_O_2_ (*v*/*v*) aqueous solution (300 mL) brought to pH 11.5 by NaOH. The mixture was stirred at 50 °C overnight and, then, centrifuged (10,000 rpm × 20 min). The supernatant (containing both lignin and hemicellulose) was first brought to pH 5.5 with 5% HCl and then 3 *V* of ethanol were added to flocculate the hemicellulose. The mixture was then filtered through a cellulose filter (Φ = 40 μm). The ethanol in the filtrate was removed by roto-evaporation and the pH lowered to 1.5–2.0 with 5% HCl to precipitate the lignin, that was dialyzed against water until Cl-free, freeze-dried, and stored for further analyses.

### 3.3. Diffuse Reflectance Infrared Fourier Transform Spectroscopy (DRIFT-IR)

DRIFT-IR spectra were recorded with a Perkin Elmer 1720-X FT-IR spectrometer (Waltham, MA, USA), equipped with a Perkin-Elmer Diffuse Reflectance accessory, by accumulating up to 8 scans with a resolution of 4 cm^−1^.

### 3.4. Solid State-Nuclear Magnetic Resonance (NMR) Spectroscopy

The ^13^C-NMR spectra of the different lignins were recorded by Cross Polarization Magic Angle Spinning (CPMAS) solid-state spectroscopy with a Bruker AVANCE 300 (Karlsruhe, Germany) using a rotating speed of 13 ± 1 kHz, a recycle time of 2 s, an acquisition time of 33 ms and 4000 scans and a contact time of 1 ms. A ^1^H ramp sequence was used to account for possible inhomogeneity of the Hartmann-Hahn condition. The free induction decays (FID) were transformed by applying a 4k zero filling and an exponential filter function with a line broadening of 100 Hz. Spectra processing was conducted by the Mestre-C software (4.9.9.9, MestreLab Research, A Coruña, Santiago de Compostela, Spain).

### 3.5. Synthesis of 2-Chloro-4,4,5,5-Tetramethyldioxaphospholane

In order to quantify the amount of different hydroxyl groups present in both lignins, a derivatizing phosphorous reagent 2-chloro-4,4,5,5-tetramethyldioxaphospholane was synthesized as described by Hatzakis *et al.* [46]. Briefly, the phosphorous reagent was obtained by mixing two solutions (A and B) prepared as follows: solution A was obtained by dissolving PCl_3_ (21.5 mL) in dry *n*-hexane (180 mL) placed in a 250 mL three-necked round flask equipped with condenser, whereas solution B was prepared by dissolving pinacol (23.7 g) in a mixture of dry pyridine (32 mL) and dry *n*-hexane (150 mL) placed in a conic flask. Solution B was added dropwise to solution A using an addition funnel properly adjusted to the second neck of the round flask for 1 h under strong stirring at 0 °C, and then the mixture was stirred for 1 h at room temperature. The white solid residue was filtered with a filter paper and rinsed with 2 × 100 mL of *n*-hexane. The solvent was removed by the rotary evaporator at 30–35 °C, and the reagent in the remaining solution was then separated by vacuum distillation (boiling point 76–78 °C at 4 mbar). The yield and purity of the product are well in line with those reported in literature [49,50,51].

### 3.6. ^31^P-Nuclear Magnetic Resonance

A stock solution composed of pyridine and deuterated chloroform in a 1.6/1 *v*/*v* ratio was added with 2.92 mg·mL^−1^ cyclohexanol, 10.0 mg·mL^−1^ of triphenyl phosphate (TPP) and 0.6 mg·mL^−1^ of chromium (III) acetylacetonate, to act as an internal standard, a reference peak for the ^31^P frequency axis calibration, and a relaxation agent, respectively. All the regent were purchased by Sigma Aldrich (Milano, Italy) Lignin samples (7.0 mg) were dissolved in 750 μL of the stock solution, and added with 50 μL of 2-chloro-4,4,5,5-tetramethyl-1,3,2-dioxaphospholane previously synthesized as ^31^P derivatization agent. Each NMR tube was ultrasonicated for 1 min prior to loading into the NMR magnet. The ^31^P-NMR spectra were recorded by an inverse gated pulse sequence, a 80 μs length (15.6 dB power level) Waltz16 scheme to decouple phosphorous from proton nuclei, a 45° pulse length of 7.8 μs and a spectral width of 400 ppm (64,935.066 Hz), where the on-resonance frequency was set to 100 ppm. 1D-Spectra consisted in a 10 s thermal delay, 304 transients, 8 dummy scans and 65,536 time domain points. The Fourier transform of Free Induction Decays (FIDs) was conducted by applying a 1.5 Hz exponential multiplication and no zero-filling. ^31^P spectra were phase and baseline corrected, while the frequency axis was calibrated by associating the value of −17.495 ppm to the TPP resonance.

### 3.7. Thermal Analysis

Thermogravimetric analysis (TGA) was carried out by air combustion of approximately 10 mg of each sample in a simultaneous thermal analyzer (STA 6000-Perkin Elmer, Waltham, MA, USA). The initial and final temperatures were 30 °C and 700 °C, respectively, with an increasing temperature rate of 10 °C·min^−1^.

### 3.8. Germination and Seedling Emergence Assays

Maize(*Zea* *mays*, L. cv. 3321 Limagrain) seeds were soaked in tap water overnight. Fifteen of the pre-soaked seeds were deposited for each replicate on round filter paper placed in a petri dish. The experiment was run in triplicates. The filters were wetted with 15 mL of lignin aqueous solutions to obtain the following treatments: 0 (control), 1, 10, 10 and 100 mg·L^−1^. Seeds were germinated in the dark at 25 °C for 96 h. Then, germination percentage was based on seeds germinated with a disrupted testa and a radicle longer than 1 mm [10]. Furthermore, the length of coleoptile, radicle and lateral seminal roots of seedlings were measured by scanning the plantlets with a Perfection V700 modified flatbed scanner (Epson, Suwa, Japan). All measurements were carried out by using the software WinRhizo version 2012b (Regent Instruments, Inc., Ch Ste-Foy, Québec, QC, Canada).

### 3.9. Statistical Analysis

The germination percentages were compared by the chi-square test, while data from the elongation experiments were treated by One-Way ANOVA and Tukey’s range test at a 95% confidence interval. All statistical tests were elaborated by using SPSS, version 21 (IBM SPPS Statistics, Armonk, NY, USA).

## 4. Conclusions

Water-soluble lignins isolated from four non-food biomasses for energy were first characterized in their molecular composition, and, then, their biological activity towards maize germination and early development was assessed. The different bioactivity of the various lignins prompted us to attempt to establish a structure-activity relationship, whose major element was the stability of the lignins’ conformational structure as a function of the hydrophobic and hydrophilic molecular components in the extracts. In particular, the most hydrophilic lignin was that isolated from cardoon, that also exerted the largest bioactivity, followed by the RIP and LIM extracts with intermediate hydrophilicity and molecular composition. The least bioactive lignin was that extracted from eucalyptus that showed the greatest hydrophobicity and, consequently, the smallest potential capacity to release bioactive molecules to the surrounding aqueous media. These findings indicate that water-soluble lignins from non-food crops and with specific molecular characteristics may be successfully employed as plant biostimulants, thus increasing the economic and ecological sustainability of biomass- and waste-based industries, such as integrated biorefineries and paper mills.

## Supplementary Materials

Supplementary materials can be accessed at: http://www.mdpi.com/1420-3049/20/11/19671/s1.
