# Supplementary material for: Water-Soluble Lignins from Different Bioenergy Crops Stimulate the Early Development of Maize (Zea mays, L.)
Source: Molecules. 2015 Nov 5;20(11):19958–70. doi: 10.3390/molecules201119671 (PMC6332221; doi:10.3390/molecules201119671)
Supplement: Supplementary file 1 [file molecules-20-19671-s001.pdf]

molecules

Supplementary Informations

Table S1. Raw data for the lengths (cm) of radicle root, lateral seminal roots and coleoptile of maize seedlings treated with different concentrations of lignins isolated from CAR.

CAR					
**Lignin Concentration (ppm)**	**Radicle Root**	**Lignin Concentration (ppm)**	**Lateral Seminal Roots**	**Lignin Concentration (ppm)**	**Coleoptile**
	6.173		5.088		2.034
	8.166		6.636		3.705
	1.1		5.212		3.279
	7.105		2.803		1.779
	1.201		6.043		5.199
	7.956		5.716		3.436
	6.081		8.588		2.695
	11.787		8.89		4.12
	11.7		9.384		6.442
	7		4.281		5.45
	12.1		4.552		3.741
0 (control)	6.601	0 (control)	10.429	0 (control)	5.335
	8.735		4.84		3.531
	11.964		10.407		4.796
	8.019		9.274		5.279
	6.109		5.111		4.425
	4.764		5.958		2.567
	4.78		5.146		2.433
	0.9		4.437		3.218
	1.036		3.025		2.702
	6.767		6.254		2.29
	5.599		1.32		3.951
	3.213		4.094		2.599
	6.585		3.66		2.557
	1.989		9.943		5.989
	6.835		8.962		3.281
	9.159		7.048		4.619
	4.402		10.685		6.922
	5.481		7.64		7.801
	6.057		7.372		7.727
	6.451		8.104		7.334
	5.717		9.697		6.993
	6.157		8.753		5.647
	4.753		9.603		6.068
	8.998		7.829		6.786
0.1	6.697	0.1	7.055	0.1	7.512
	8.499		8.754		7.258
	5.15		5.705		5.147
	5.714		5.034		5.624
	4.452		1.84		4.834
	4.449		2.915		2.15
	1.798		3.299		4.293
	1.501		2.992		3.339
	4.774		2.809		4.159
	1.662		6.07		4.028
	9		4.096		3.721
	1.66		3.687		4.328
	7.53		12.35		6.29
	7.58		3.95		5.937
1	7.34	1	6.92	1	6.233
	10.91		10.74		6.184
	5.63		6.64		5.49

	12.01		7.14		6.12
	11.65		11.31		6.213
	4.27		3.66		6.569
	5.81		5.47		7.267
	6.34		5.7		7.245
	1.1		6.27		5.193
	5.38		5.85		4.055
	6.43		9.41		3.122
	5.13		6.78		4.408
	4.56		5.88		4.495
	6.64		8.24		3.941
	8.32		4.22		5.384
	7.4		6.81		4.011
	6.92		11.93		6.127
	1.09		6.62		2.573
	6.01		4.23		2.391
	5.91		7.27		4.833
	0.99		2.87		6.093
	5.58		3.11		5.883
	6.59		3.23		5.838
	0.5		3.66		6.213
	6.33		5.19		6.304
	6.07		5.35		3.567
	0.39		5.44		2.126
	0.8		6.47		7.016
	6.7		6.9		4.535
	6.89	10	6.91		5.95
	7.78		7.26		7.074
	12.001		8.49		6.313
10	8.68		9.24		5.679
	8.82		12.27		3.474
	7.59		12.43		9.116
	12.411		12.93		8.968
	12.001		13.21		4.575
	6.7		13.24	10	3.893
	8.01		13.29		6.288
	8.61		7.02		6.549
	5.35		4.67		6.818
	6.31		3.46		8.46
	11.84		6.96		7.537
	6.09		5.39		6.944
	6.27		1.1		5.076
	4.95		4.94		6.872
	3.97		3.59		7.957
	0.746		6.2		7.402
	5.2		8.96		7.645
	4.64		6.62		7.6
	1.242		3.63		4.598
	0.717	100	5.19		6.984
	8.5		5.22		5.411
	5.11		3.85		5.995
100	4.89		4.28		6.739
	0.654		10.03		5.218
	1.302		6.95		3.217
	5.01		5.44		3.381
	3.94		5.08	100	3.853
	4.31		4.74		3.341
	5.51		4.9		4.456
	9.146		6.43		4.242
	6.28		7.64		4.457
	4.7		11.06		4.468
	9.056				3.929

	6.3				3.66
	8.56				4.749
	4.31				4.284
	9.003				6.224
	4.17				3.575
					4.939
					5.354
					6.401
					4.808
					4.381
					4.329

Table S2. Raw data for the lengths (cm) of radicle root, lateral seminal roots and coleoptile of maize seedlings treated with different concentrations of lignins isolated from EUC.

EUC					
**Lignin concentration (ppm)**	**Radicle root**	**Lignin concentration (ppm)**	**Lateral Seminal Roots**	**Lignin concentration (ppm)**	**Coleoptile**
0 (control)	7.4	0 (control)	14.2	0 (control)	7.44
	7.75		16.05		8.14
	8.85		18.98		8.36
	9.16		21.01		7.31
	9.43		21.74		8.68
	9.89		23.15		8.89
	10.97		23.26		7.41
	11.69		24.48		9.12
	7.11		6.34		9.31
	7.73		8.62		7.68
	9.49		11.34		7.79
	9.85		14.3		8.13
	10.1		22.52		8.32
	11.14		23.5		8.56
	12.73		24.79		8.98
	11.02		16.79		8.98
	11.32		19.83		8.31
	11.46		20.49		8.34
	12.65		21.08		8.34
	12.86		21.68		8.47
	13.58		22.43		8.48
	13.8		23.97		8.9
	14.51		25.29		9.46
	14.77		28.94		9.52
0.1	8.03	0.1	14.25		9.54
	8.94		16.19		9.56
	9.57		18.79	0.1	8.27
	10.38		18.88		8.41
	11.67		19.44		8.58
	11.75		20.07		8.91
	11.87		20.46		9.15
	12.28		21.23		9.18
	12.62		24.59		9.33
1	6.61	1	16.13		9.43
	9.52		16.44	1	7.45
	9.52		17.02		7.81
	9.54		17.37		8.58
	11.95		18.59		9.03
	12		18.69		9.15
	12.17		20.28		9.3
					9.55
	13.8		24.68		9.89
10	6.61	10	5.58	10	8.73
	6.85		10.75		8.69

	8.75		11.13		2.51
	9.26		11.47		9.28
	9.26		16.97		9.48
	9.76		17.39		9.29
	9.94		18.5		8.81
	11.91		23.87		8.85
	13.42		28.54		7.64
100	8.1	100	14.45	100	9.48
	8.59		14.76		6.88
	9.19		20.94		7.48
	9.32		21.21		7.78
	9.62		22.23		7.92
	9.66		23.91		8.16
	11.62		24.83		8.66
	13.62		25.33		8.67
	9.15		21.23		7.9
	15.07		22.88		7.91
	6.99		6.34		8.33
	11.97		22.5		8.38
	6.97		22.07		8.4
	10.6		26.22		8.83
	13.61		24.02		8.92
	11.4		23.97		9.33
	12.99		27.24		9.4
	11.31		28.82		7.71
	12.47		22.51		7.76
	13.87		16.42		7.86
	11.98		19.29		8.22
	9.17		20.89		8.32
	6.49		9.04		8.37
	7.34		26.03		8.49
	9.86		11.03		9.21
	11.3		21.6		9.22

Table S3. Raw data for the lengths (cm) of radicle root, lateral seminal roots and coleoptile of maize seedlings treated with different concentrations of lignins isolated from RIP.

RIP					
Lignin concentration (ppm)	Radicle root	Lignin concentration (ppm)	Lateral Seminal Roots	Lignin concentration (ppm)	Coleoptile
0 (control)	6.339	0 (control)	2.126	0 (control)	2.077
	3.191		2.25		2.228
	4.742		2.85		2.484
	7.134		3.129		2.676
	6.917		4.454		2.976
	3.752		5.282		3.605
	2.439		5.419		4.52
	6.267		6.005		4.815
	6.165		6.044		4.854
	4.913		6.084		4.982
	3.693		6.566		5.238
	3.717		6.959		5.294
	2.919		7.028		5.459
	2.767		8.076		5.601
	4.938		8.635		5.61
	5.749		8.971		5.625
	6.379		9.735		5.675
	4.61		9.742		5.714
	5.143		10.101		5.734
	5.483		10.431		5.751
	4.019		10.735		5.927

	3.347
	8.583
	7.042
	7.49
	5.872
	6.813
	9.2
	3.193
	8.216
	8.682
	6.688
0.1	3.714
	9.73
	10.535
	6.944
	6.825
	5.093
	8.169
	6.164
	7.957
	7.02
	7.064
	9.19
	7.442
	3.126
	6.373
	9.237
	8.383
	6.456
	3.373
	3.252
	6.763
	8.459
	7.531
	7.531
	6.601
	7.07
	8.112
	8.382
	8.112
	3.64
	4.875
	5.657
	4.76
	4.865
	6.193
1	6.213
	4.948
	6.963
	5.245
	7.204
	5.93
	6.024
	6.601
	6.359
	6.467
	3.633
	3.731
	5.742
	6.865
	6.084
	5.964

	10.823
	11.308
	11.761
	12.051
	12.067
	12.932
	13.577
	14.001
0.1	4.579
	6.6
	6.862
	7.331
	7.814
	8.015
	8.498
	8.611
	8.781
	8.909
	8.967
	8.991
	9.393
	9.627
	9.944
	9.972
	11.102
	11.297
	11.346
	11.447
	11.519
	11.959
	12.083
	12.365
	14.247
	14.458
	14.681
	15.314
	15.46
	15.658
	15.968
	18.002
	18.879
1	3.394
	3.563
	3.799
	4.571
	5.052
	5.429
	5.676
	6.121
	7.272
	7.406
	7.645
	7.678
	7.884
	7.966
	8.533
	9.011
	9.383
	9.517
	9.583
	9.664
	10.114

	5.967
	6.012
	6.024
	6.208
	6.266
	6.543
	6.58
	6.674
	6.776
0.1	4.147
	4.2
	4.526
	4.537
	4.711
	4.718
	4.722
	4.751
	4.958
	5.12
	5.141
	5.156
	5.267
	5.307
	5.417
	5.419
	5.426
	5.428
	5.517
	5.58
	5.585
	5.745
	5.763
	5.809
	5.888
	5.932
	5.979
	5.979
	6.091
	6.242
	6.468
	6.62
1	4.212
	4.39
	4.449
	4.78
	5.001
	5.175
	5.214
	5.262
	5.378
	5.407
	5.412
	5.47
	5.476
	5.6
	5.697
	5.717
	5.745
	5.83
	5.843
	5.855
	5.859

	4.018
	5.081
	5.163
	4.631
	5.01
	6.638
	7.558
	8.096
	6.986
	5.358
	5.781
	5.879
	6.991
	6.717
	6.385
	7.876
	7.008
	6.017
	5.875
	6.395
	5.912
	4.563
	5.509
	3.654
	7.752
	7.453
	5.427
	5.196
10	6.887
	8.604
	5.987
	6.279
	6.109
	5.917
	3.622
	7.786
	6.544
	7.811
	8.377
	9
	5.47
	7.29
	8.276
	4.359
	7.753
	5.238
	6.335
	6.232
	7.086
	5.427
	7.266
	6.623
100	10.685
	7.03
	8.764
	9.106
	9.408
	9.159
	3.172
	8.945
	8.952
	7.776

	10.654
	10.684
	11.344
	11.758
	12.072
	3.025
	4.289
	6.141
	6.203
	6.427
	7.037
	7.616
	7.829
	7.83
	7.953
	8.234
	8.966
	9.054
10	9.382
	9.693
	9.989
	10.58
	10.599
	11.133
	11.707
	11.994
	12.785
	13.791
	13.966
	14.377
	15.108
	15.344
	15.843
	4.014
	4.416
	4.468
	5.138
	5.475
	6.247
	6.494
	6.814
	6.859
	7.402
	8.562
	8.847
	9.811
	10.089
100	10.841
	10.849
	11.541
	12.066
	12.156
	12.366
	13.022
	13.43
	13.841
	14.227
	14.258
	14.38
	14.956
	16.251
	16.402

	5.86
	5.874
	5.898
	6.139
	6.188
	6.225
	6.476
	3.041
	3.21
	3.447
	3.781
	3.878
	4.153
	4.409
	4.493
	4.605
	4.715
	5.007
	5.122
	5.434
	5.634
	5.64
	5.662
	5.717
10	5.72
	5.789
	5.962
	5.981
	6.139
	6.145
	6.222
	6.265
	6.45
	6.651
	6.67
	6.833
	6.923
	6.957
	7.008
	7.103
	7.162
	7.228
	3.188
	3.308
	3.672
	4.395
	4.529
	4.696
	4.781
	4.821
	4.931
100	5.25
	5.335
	5.572
	5.733
	5.936
	6.187
	6.403
	6.537
	6.542
	6.572
	6.618

	6.8				6.638
	9.804				6.709
	3.376				6.71
	4.06				6.771
	7.475				6.784
	5.464				6.957
	8.127				7.023
	4.884				7.034
	3.575				7.048
	3.194				7.054
	6.831				7.906
	4.312				
	8.551				

Table S4. Raw data for the lengths (cm) of radicle root, lateral seminal roots and coleoptile of maize seedlings treated with different concentrations of lignins isolated from LIM.

LIM					
**Lignin concentration (ppm)**	**Radicle root**	**Lignin concentration (ppm)**	**Lateral Seminal Roots**	**Lignin concentration (ppm)**	**Coleoptile**
0 (control)	7.298	0 (control)	2.704	0 (control)	4.426
	5.797		7.818		2.999
	6.554		11.258		2.645
	4.99		6.955		4.664
	5.845		7.232		6.025
	6.302		9.799		5.861
	7.171		7.948		4.569
	6.14		8.518		5.416
	7.738		8.163		4.188
	4.431		6.002		5.276
	6.93		10.963		3.93
	3.619		5.975		6.351
	3.601		8.623		3.415
	6.054		6.092		3.652
	6.413		10.615		4.604
	6.819		8.676		5.921
	7.92		10.309		4.877
	7.266		12.952		5.851
	5.818		6.46		4.431
	6.638		6.077		5.56
	6.098		8.224		6.04
	3.41		5.758		5.474
	5.734		11.137		6.417
	9.643		15.789		6.105
	6.613		9.554		6.117
	7.405		9.985		6.393
	7.198		9.674		4.528
	8.682		7.316		7
	7.208		6.284		5.942
	7.737		6.241		4.966
	6.952		9.853		5.135
	3.629		15.321		5.279
0.1	9.695	0.1	7.924		5.853
	3.386		10.432		6.102
	4.319		9.419	0.1	5.777
	9.065		2.219		5.369
	8.855		15.054		5.81
	10.072		10.178		6.196
	6.064		3.929		6.393
	6.49		10.247		6.45
	6.991		14.689		6.362
	3.069		10.504		6.016

	4.164		14.723		4.792
	5.94		7.467		7.216
	6.877		11.326		6.111
	7.366		9.186		6.322
	8.202		10.426		4.543
	5.757		6.139		4.454
	7.283		12.989		6.061
	7.096		6.821		6.175
	5.239		8.161		5.783
	5.803		6.795		5.849
	6.966		6.17		4.597
	4.053		9.205		4.428
	6.876		6.853		5.606
	7.345		9.307		5.975
	6.809		9.094		5.708
	4.421		3.709		4.175
	7.108		6.304		5.355
	5.76		7.637		6.112
	4.974		7.614		5.351
	6.665		12.539		6.066
	8.193		7.521		6.003
	7.472		15.916		4.702
	9.81		4.498		6.2
	7.846		9.416		6.742
	9.028		13.452		5.463
	3.792		5.285		5.32
	5.838		6.964		4.79
	6.234		6.823		3.863
	7.726		8.248		4.261
	4.906		12.451		5.381
	5.035		16.024		6.726
	7.263		9.369		6.786
	9.439		11.071		5.267
	8.499		16.729		4.742
1	5.55	1	4.301		7.005
	4.662		13.678		4.953
	6.39		15.884		5.575
	6.671		10.26		5.431
	9.21		5.932		6.258
	8.271		5.238		5.51
	9.318		8.883		5.751
	8.574		6.073		5.466
	8.91		9.468	1	5.054
	5.018		13.143		5.86
	6.352		10.41		5.709
	9.175		6.825		4.95
	8.241		11.959		4.94
	7.958		16.342		5.922
	7.692		12.88		5.509
	8.581		11.886		4.168
	9.533		14.486		6.179
	5.195		6.08		6.748
	7.366		6.666		5.254
	9.025		6.007		6.066
	6.533		6.751		5.204
10	9.462	10	7.872		5.328
	7.862		9.316		5.136
	4.235		4.242		6.254
	7.632		13.246		5.487
	6.29		11.364		6.281
	8.844		3.687	10	5.632
	5.632		10.271		5.356

	7.186		9.108		5.869
	5.906		9.394		4.608
	4.416		14.322		5.985
	5.894		13.28		4.444
	3.999		4.614		6.445
	3.214		5.427		4.937
	4.019		6.341		4.477
	4.974		11.696		5.084
	5.632		11.101		4.805
	8.568		12.086		6.977
	3.446		8.803		5.853
	5.423		15.886		4.833
	6.473		11.564		4.683
	7.727		6.302		5.295
	5.889		12.957		4.904
	6.005		13.412		4.99
	4.347		6.217		5.297
	7.357		6.507		5.07
	7.678		10.382		5.341
	9.946		14.262		5.605
	9.469		10.414		5.506
	9.507		10.273		5.27
	10.124		12.05		6.002
	9.366		10.146		5.846
	6.35		3.233		4.67
	4.73		11.501		5.095
	10.7		9.005		6.455
	9.152		8.856		6.527
	10.401		10.214		3.877
	7.217		7.816		7.217
	9.631		8.934		5.684
	7.183		4.807		6.033
	7.703		8.236		6.594
	4.626		7.94		5.623
100	9.55	100	2.338		5.781
	8.173		8.674		6.844
	5.755		4.266		5.987
	9.456		11.683		6.358
	4.03		14.05		6.089
	9.126		4.572	100	4.699
	5.585		8.748		7.308
	7.555		5.87		6.877
	7.731		7.175		6.521
	7.762		12.799		5.458
	6.315		3.343		7.134
	7.26		9.426		5.833
	7.637		9.318		6.231
	2.174		8.456		6.038
	4.096		11.869		5.702
	4.537		12.8		5.517
					5.492
					6.66
					3.989
					6.343
					7.488
